# Supplementary material for: Maternal, fetal, and perinatal outcomes among pregnant women admitted to an Ebola treatment center in the Democratic Republic of Congo, 2018–2020
Source: PLoS One. 2023 Sep 8;18(9):e0286843. doi: 10.1371/journal.pone.0286843 (PMC10490991; doi:10.1371/journal.pone.0286843)
Supplement: Attachment — Submitted filename: Reviewer_Replies.docx [file pone.0286843.s001.docx]

Introduction and rationale: this was well written and provided enough background information justifying the study.

Thank you.

Methods: the methods are succinct and well described.

Thank you.

Results: the results are clear and presented in full.

Thank you.

Discussion and conclusions: while the discussion supports the results, I would like the authors to elaborate a little more on possible reasons for the mortality relative risk of 1.0. The study objectives indicate you wish to compare mortality in pregnant and non-pregnant women and while your findings are interesting and supported both by a recent systematic review Kayem et al 2022 and some studies from the West African Ebola outbreak, you do not highlight these and right now your discussion on this finding is a single sentence at the end of your discussion section. I would like you to give us your opinion on possible reasons why no difference in mortality is found.

Additionally based on the findings in your study, what are your recommendations for practice, research and policy.

Thank you for your requested clarification. We have expanded our discussion with additional consideration for why we did not identify an increased risk of death among pregnant patients in the study and included a reference to the Kayem et al review as well as data from the West Africa Outbreak. Likewise, we have expanded recommendations for future data collection to include pregnant women as an important subgroup.
